# Temporal Changes in Finger and Upper Limb Usage During Hospitalization After Distal Radius Fracture Surgery: A Case Report

**DOI:** 10.7759/cureus.100901

**Published:** 2026-01-06

**Authors:** Naoya Yamamoto, Yasuhiro Kitagawa, Megumi Miyashita, Toshiyuki Kondo

**Affiliations:** 1 Department of Rehabilitation, Shonan Keiiku Hospital, Fujisawa, JPN; 2 Department of Orthopedic Surgery, Shonan Keiiku Hospital, Fujisawa, JPN; 3 Institute of Engineering, Tokyo University of Agriculture and Technology, Tokyo, JPN

**Keywords:** distal radius fracture, finger usage, postoperative rehabilitation, sensing, wearable device

## Abstract

Recovery after distal radius fracture surgery is commonly evaluated using physical function measures; however, actual upper limb use in daily life may not be fully captured by these assessments. In this case report, a postoperative distal radius fracture patient was monitored during hospitalization using a ring-shaped wearable device to quantitatively assess finger and upper limb usage, together with patient-reported outcomes and physical function measures. Finger and upper limb usage ratios increased during the mid-postoperative period, accompanied by reductions in swelling and improvements in the Japanese version of the Patient-Rated Wrist Evaluation scores, whereas improvements in range of motion and grip-related function occurred earlier in the postoperative course. This case describes the temporal relationship between physical function, symptoms, and objectively measured upper limb usage during hospitalization, and shows that changes in actual upper limb usage occurred concurrently with reductions in swelling and pain.

## Introduction

Distal radius fracture is one of the most common fractures among the elderly [1], and patients with osteoporosis are particularly prone to sustaining such fractures even from minor trauma. Open reduction and internal fixation (ORIF) using a volar locking plate, one of the treatment options, has been reported to provide excellent outcomes in terms of bone union and maintenance of reduction [2], and its indications have expanded in recent years. However, despite achieving anatomical reduction, a considerable number of patients still experience long-term functional impairments after surgery. A report on patients with traumatic hand injuries indicated that approximately 90% continued to experience moderate or greater difficulties in daily life [3], suggesting that similar problems may also occur in patients with distal radius fractures.

Traditional rehabilitation has primarily focused on the recovery of physical functions such as joint range of motion and muscle strength. However, it has already been pointed out in the field of stroke rehabilitation that improvement in “capacity” does not necessarily guarantee actual “performance” [4]. In patients after a distal radius fracture, factors such as pain, swelling, and fear of movement may hinder the active use of the affected upper limb, potentially delaying functional recovery. In particular, pain-related avoidance behaviors and experiences of failure are suggested to induce learned non-use, further reducing the use of the affected limb [5].

In addition, there have been limitations in methods for objectively assessing the amount of finger and upper limb use. Traditionally, evaluations have relied on self-reports or observational assessments, but these are subject to subjective bias, making it difficult to accurately capture actual usage. In this regard, recent studies in stroke patients have demonstrated the effectiveness of monitoring upper limb use with accelerometers and wearable devices [4], and such technologies are gaining attention as potentially applicable to postoperative orthopedic patients [6]. However, reports applying these objective evaluations to patients after distal radius fracture surgery are extremely limited. A systematic review by Roll et al. [7] also highlighted that interventional studies at the activity and participation levels for the forearm, wrist, and hand regions are very scarce, further underscoring the importance of quantitatively assessing upper limb use in real-life contexts.

Based on the above background, the present case report aimed to continuously measure the amount of finger and upper limb use during hospitalization in a postoperative distal radius fracture patient using a ring-shaped wearable device, and to examine the relationship between its temporal changes and improvements in functional impairment. In this study, temporal effects refer to the time-dependent changes in symptoms, physical function, and actual finger and upper limb usage observed during the postoperative course.

## Case presentation

Patient

**Figure 1:**
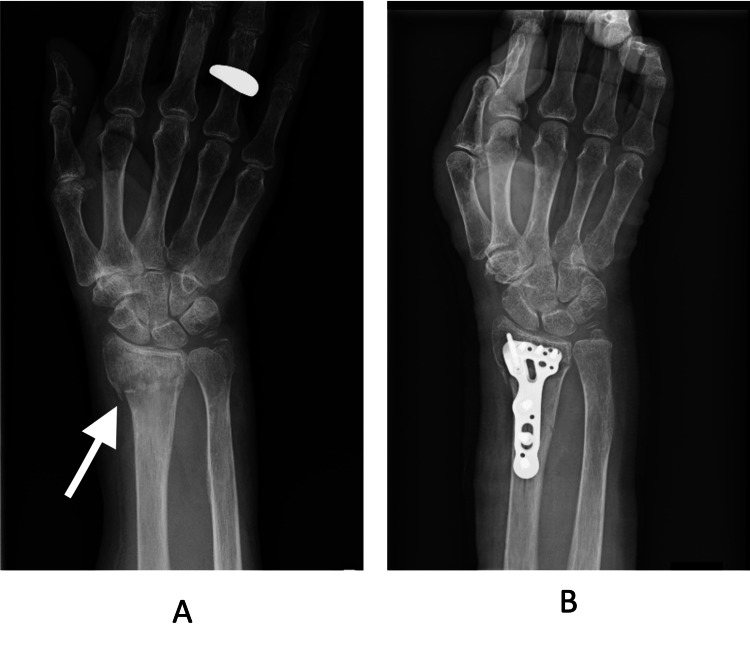
Preoperative and postoperative radiographs of the distal radius fracture. Radiographs of the right wrist in a patient with a distal radius fracture (AO classification A2). (A) Preoperative radiograph. (B) Postoperative radiograph obtained on the day of surgery after open reduction and internal fixation using a volar locking plate.

The patient was a right-handed female in her late 70s who had been independent in her activities of daily living (ADL) prior to the injury. She had a medical history of hypertension. The diagnosis was a right distal radius fracture (AO classification A2), sustained due to a fall while walking outdoors. ORIF using a volar locking plate was performed three days after the injury, and physical therapy was initiated on postoperative day one. She was discharged home on postoperative day 22. Preoperative radiographs and radiographs obtained on the day of surgery after ORIF are shown in Figure 1.

Evaluation period

Evaluations were conducted three times: on postoperative day seven (PO7), postoperative day 14 (PO14), and postoperative day 21 (PO21). The period from PO7 to PO14 was defined as phase A, and the period from PO14 to PO21 was defined as phase B. Changes observed within each phase were analyzed. During hospitalization, the patient received physical therapy for approximately 20 minutes per day. The rehabilitation program included range of motion exercises for the wrist and fingers, edema management, and practice of functional upper limb use in daily activities. Although the difficulty of tasks was adjusted according to pain levels, the main content and overall intensity of the program remained consistent throughout the hospital stay.

Evaluation items and measurement methods

**Figure 2:**
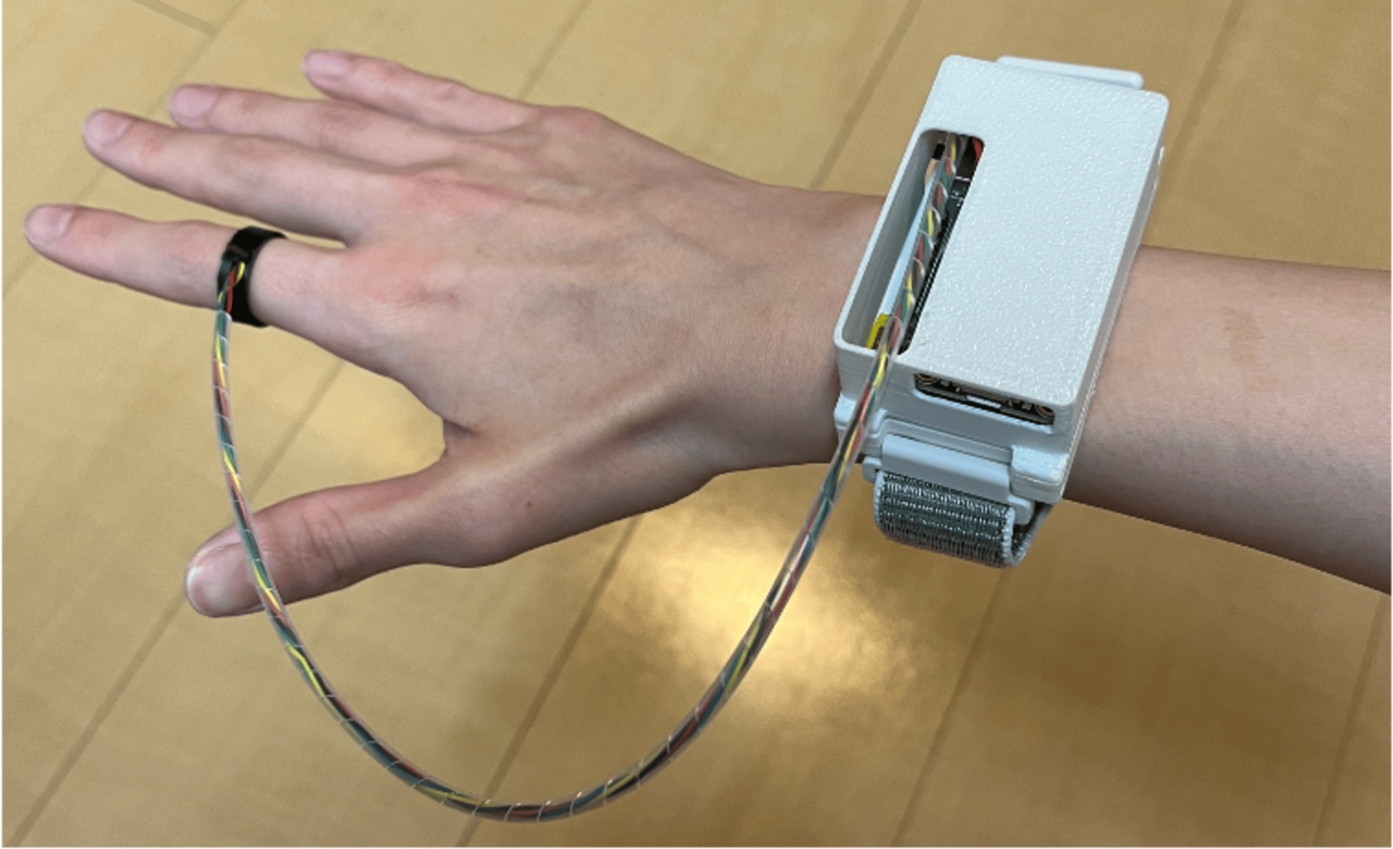
Ring-shaped wearable device used to measure finger and upper limb usage. Ring-shaped wearable device equipped with an infrared distance sensor and an accelerometer, used to quantitatively measure finger and upper limb usage on both the operated and non-operated sides. The weight of this device is 35 g.

A ring-shaped wearable device equipped with an infrared distance sensor and an accelerometer [8] was used to measure the amount of finger and upper limb usage on both the operated and non-operated sides, as shown in Figure 2.

The device consists of a light-emitting diode (LED; SFH4550, Osram, Munich, Germany) and a phototransistor (SD5410, Honeywell, Charlotte, NC) and was worn on the proximal phalanx of the index finger. A microcontroller (Adafruit Feather M0 Adalogger, Adafruit, New York City, NY) and a rechargeable lithium-polymer battery (3.7 V, 400 mAh) were housed in a wrist-mounted enclosure. The distance between the fingertip and the ring device, d(n), was recorded at 100 Hz. Using the pre-measured distance between the fingertip and the proximal interphalangeal (PIP) joint, L, the PIP joint flexion angle θ(n) was calculated as θ(n) = arccos (d(n)/L), where n represents time steps sampled at 10-ms intervals. The accumulated change in joint angle was defined as finger usage, and the cumulative norm of acceleration obtained from the wrist-mounted accelerometer was used as an index of upper-limb usage.

Measurements were conducted on PO7, PO14, and PO21, and the total amount of usage during a 12-hour period (8:00-20:00) on each evaluation day was recorded. The finger usage ratio was defined as the ratio of finger usage on the operated side to that on the non-operated side. Similarly, the upper limb usage ratio was defined as the ratio of upper limb usage on the operated side to that on the non-operated side.

The maximum range of motion of the wrist (dorsiflexion, palmar flexion, radial deviation, and ulnar deviation) as well as forearm pronation and supination was measured using a goniometer. Measurements were conducted in accordance with the standard anatomical position and within a range that did not cause discomfort to the patient. In addition, as an indicator of finger function, the tip-palm distance (TPD), defined as the distance between the tip of the index finger and the palm during gripping, was measured.

Swelling of the operated-side forearm and index finger was assessed. Forearm circumference was measured at a point 3 cm proximal to the radial styloid process, and index finger circumference was measured at the midpoint between the second metacarpophalangeal joint and the PIP joint. A non-elastic tape measure was used in all cases, avoiding excessive compression during measurement.

As a subjective assessment of impairment in the operated wrist, the Japanese version of the Patient-Rated Wrist Evaluation (PRWE-J) [9], originally developed by MacDermid [10], was used with permission from the original developer. The PRWE-J consists of two subscales: a pain subscale (PRWE-P) and a function subscale (PRWE-F), and the assessment was conducted in a self-administered format by the patient. The PRWE-P includes five items assessing wrist pain intensity during different activities, with each item scored from 0 (no pain) to 10 (worst pain imaginable), yielding a total score ranging from 0 to 50. Higher PRWE-P scores indicate greater pain-related impairment. The PRWE-F includes 10 items assessing difficulty in performing specific functional tasks and usual activities involving the wrist. Each item is scored from 0 (no difficulty) to 10 (unable to perform), resulting in a total score ranging from 0 to 50. Higher PRWE-F scores indicate greater functional limitation. All PRWE-J assessments were performed according to the original scoring instructions.

Ethical considerations

This case report was conducted with the approval of the Ethics Committee of Shonan Keiiku Hospital (19-002). It was prepared in accordance with the CARE (Case Report) guidelines, an international standard for case reports. Prior to conducting the study, the patient was provided with sufficient explanation regarding the purpose, methods, and privacy protection measures of the study, both verbally and in writing, and written informed consent was obtained.

Results

The evaluation results are presented in the order of PO7, POD 14, and POD 21 to illustrate whether greater improvement trends were observed in phase A or phase B. Table 1 summarizes the temporal changes in finger usage ratio, upper limb usage ratio, range of motion, swelling, and PRWE-J scores across the postoperative phases.

**Table 1:** Summary of outcome measures. PO: postoperative day (after surgery); ROM: range of motion; TPD: tip-palm distance; PRWE-J: Japanese version of the Patient-Rated Wrist Evaluation.

Outcome measures		PO7	PO14	PO21
Finger usage ratio		0.18	0.28	0.64
Upper-limb usage ratio		0.24	0.41	0.79
ROM	Dorsiflexion (°)	20	40	45
	Palmar flexion (°)	30	45	50
	Radial deviation (°)	5	5	5
	Ulnar deviation (°)	20	25	30
	Pronation (°)	80	90	90
	Supination (°)	30	75	90
	TPD (cm)	2	0	0
Swelling	Forearm circumference (cm)	18	17.7	16.9
	Index finger circumference (cm)	7.8	7.5	6.8
PRWE-J	Pain (points)	42	33	8
	At rest	6	3	0
	When doing a task with a repeated wrist movement	9	7	1
	When lifting a heavy object	9	8	2
	When it is at its worst	9	8	3
	How often do you have pain?	9	7	2
	Function (points)	48	39	17.5
	Turn a doorknob using my affected hand	9	8	1
	Cut meat using a knife with my affected hand	10	9	4
	Fasten the buttons on my shirt	10	8	2
	Use my affected hand to push up from a chair	9	8	4
	Carry a 10 lb object with my affected hand	10	8	5
	Use bathroom tissue with my affected hand	9	7	4
	Personal care activities (dressing, washing)	9	7	2
	Household work (cleaning, maintenance)	10	8	5
	Work (your job or usual everyday work)	10	8	5
	Recreational activities	10	7	3

The finger usage ratio increased from 0.18 to 0.28 and further to 0.64, while the upper limb usage ratio increased from 0.24 to 0.41 and then to 0.79. Both ratios showed a greater increase during phase B compared with phase A.

Regarding range of motion, wrist dorsiflexion increased from 20° to 40° and 45°, and palmar flexion increased from 30° to 45° and 50°. Ulnar deviation increased from 20° to 25° and 30°, whereas radial deviation remained unchanged at 5°. Pronation improved from 80° to 90° and remained stable thereafter, while supination increased from 30° to 75° and 90°. The TPD improved from 2 cm to 0 cm by phase A and remained unchanged thereafter, indicating that improvements in range of motion occurred mainly during phase A.

Swelling of the forearm decreased from 18.0 cm to 17.7 cm and 16.9 cm, and swelling of the index finger decreased from 7.8 cm to 7.5 cm and 6.8 cm. These reductions were more pronounced during phase B.

The PRWE-J scores also showed improvement over time. The PRWE-P decreased from 42 to 33 and then to 8, and the PRWE-F decreased from 48 to 39 and then to 17.5, with greater improvements observed during phase B. No adverse or unanticipated events occurred during the clinical course.

## Discussion

In this case report, finger and upper limb usage were quantitatively measured in a postoperative distal radius fracture patient using a ring-shaped wearable device, and temporal changes in usage during the postoperative course were described. The finger usage ratio and upper limb usage ratio showed a greater increase in the mid-postoperative phase (phase B) than in the early postoperative phase (phase A). During phase B, reductions in swelling and concurrent improvements in both the PRWE-P and PRWE-F were observed. In contrast, the range of motion and the TPD, an indicator of gripping function, mainly improved during phase A. These findings indicate that changes in finger and upper limb usage, pain, and swelling occurred during overlapping periods in this case.

The increases in finger and upper limb usage ratios observed during phase B occurred during the same period as reductions in pain and swelling. In the early postoperative period, local inflammatory responses commonly result in swelling and pain, which may suppress spontaneous use of the affected upper limb. Pain has been reported to act as both a psychological and physiological barrier to voluntary upper limb movement [5]. Lang et al. [4] similarly reported that pain and experiences of movement failure may be associated with reduced upper limb usage in patients with post-stroke hemiparesis. In this case, reductions in forearm and index finger swelling during phase B were observed alongside improvements in PRWE-P and PRWE-F scores, suggesting a temporal association between symptom improvement and increased upper limb usage. In addition, Buchman et al. [11] reported that musculoskeletal pain in older adults is associated with an increased risk of developing disabilities in activities of daily living. In light of these findings, the concurrent changes observed in pain, swelling, and finger and upper limb usage in this case may be interpreted within a broader framework in which pain-related symptoms are related to actual upper limb use in daily life. In addition, this ring-shaped wearable device has previously been used to quantify finger and upper limb usage in patients with stroke, demonstrating its feasibility for objectively assessing upper limb performance in daily life [8]. The present case extends these findings to a postoperative orthopedic context, suggesting potential applicability of this device across different upper limb conditions.

PRWE is a patient-reported outcome measure assessing pain and functional impairment. Improvement in PRWE-P reflects a reduction in pain-related activity limitations, while improvement in PRWE-F may reflect increased active use of the affected limb in activities of daily living [10]. Therefore, changes in PRWE scores observed in this case may be related to changes in finger and upper limb usage.

Although the range of motion and TPD mainly improved during phase A, a marked increase in finger or upper limb usage ratios was not observed during this period. Improvements were observed in wrist dorsiflexion, palmar flexion, ulnar deviation, pronation, and supination, and TPD normalized during phase A. However, these improvements in physical function were not accompanied by a corresponding increase in actual upper limb usage in daily life. As noted by Roll et al. [7], improvements in physical function alone do not necessarily translate into gains at the activity or participation level. A similar tendency was observed in this case.

Overall, this case highlights differences in the timing of changes in physical function, symptoms, and upper limb usage after distal radius fracture surgery. The findings suggest that postoperative recovery may involve multiple factors beyond physical function alone, and that actual upper limb usage in daily life may change differently from physical impairment measures. From a clinical perspective, monitoring real-world upper limb use may help identify gaps in postoperative recovery that are not fully captured by conventional assessments such as range of motion or TPD alone.

This study has several limitations. The first limitation of this case report is that the observed increase in finger and upper limb usage and the reduction in pain and swelling were temporally associated, but this temporal association does not establish a causal relationship. Although improvements in pain and swelling coincided with increased usage in this case, it cannot be concluded that reductions in pain and swelling directly led to increased finger and upper limb usage, and this interpretation should be made with caution. Second, the observation period was relatively short, limited to 21 days after surgery, which prevented evaluation of medium- to long-term changes in finger and upper limb usage and their potential impact on activities of daily living and quality of life. Third, psychological and social factors other than pain and swelling, such as fear-avoidant behaviors, motivation, and self-efficacy, were not sufficiently assessed and may have influenced upper limb usage. In addition, other factors such as the patient’s medical history and rehabilitation procedures may also have acted as potential confounders. Future studies incorporating longer follow-up periods and comprehensive assessments are needed to better understand the factors associated with postoperative upper limb use.

In the future, longitudinal studies involving multiple cases are needed to examine the causal relationship between the reduction of pain/swelling and improvements in finger and upper limb usage, as well as to clarify the effectiveness of comprehensive rehabilitation interventions, including pain management. In particular, it will be important to verify the extent to which early postoperative interventions focusing on inflammation control contribute to the natural promotion of upper limb usage.

## Conclusions

In this case, finger and upper limb usage increased during the postoperative course after distal radius fracture surgery, while improvements in range of motion were observed earlier. Changes in pain and swelling occurred alongside changes in actual upper limb usage. Although based on a single case, this report describes the temporal relationship between physical function, symptoms, and objectively measured upper limb usage during hospitalization. Objective assessment of finger and upper limb usage using wearable devices may help characterize postoperative recovery at the performance level.
